# Development of an Indirect ELISA and Dot-Blot Assay for Serological Detection of Rice Tungro Disease

**DOI:** 10.1155/2017/3608042

**Published:** 2017-10-22

**Authors:** Magdline Sia Henry Sum, Siew Fung Yee, Lily Eng, Evenni Poili, Julia Lamdin

**Affiliations:** ^1^Institute of Health & Community Medicine, Universiti Malaysia Sarawak, 94300 Kota Samarahan, Sarawak, Malaysia; ^2^Agriculture Research Centre (ARC), Department of Agriculture (DOA) Sarawak, Semongok, Borneo Heights Road, 93250 Kuching, Sarawak, Malaysia; ^3^Agriculture Research Centre (ARC), Department of Agriculture (DOA) Sabah, P.O. Box No. 3, 89207 Tuaran, Sabah, Malaysia; ^4^Agriculture Research Centre (ARC), Department of Agriculture Sabah, P.O. Box No. 2050, 88632 Kota Kinabalu, Sabah, Malaysia

## Abstract

Rice tungro disease (RTD) is one of the most destructive diseases of rice in South and Southeast Asia. RTD is routinely detected based on visual observation of the plant. However, it is not always easy to identify the disease in the field as it is often confused with other diseases or physiological disorders. Here we report the development of two serological based assays for ease of detection of RTD. In this study we had developed and optimized an indirect ELISA and dot-blot assay for detection of RTD. The efficiency of both assays was evaluated by comparing the specificity and sensitivity of the assays to PCR assay using established primer sets. The indirect ELISA showed 97.5% and 96.6%, while the dot-blot assay showed 97.5% and 86.4% sensitivity and specificity, respectively, when compared to established PCR method. The high sensitivity and specificity of the two assays merit the use of both assays as alternative methods to diagnose RTD. Furthermore, the dot-blot assay is a simple, robust, and rapid diagnostic assay that is suitable for field test for it does not require any specialized equipment. This is a great advantage for diagnosing RTD in paddy fields, especially in the rural areas.

## 1. Introduction

Rice tungro disease (RTD), which causes reduction in rice production, is a widespread viral disease in South and Southeast Asia. In one of the worst reported outbreaks, it was estimated to cause annual losses in excess of about US$1.5 × 109 [1]. The disease is caused by infection of two different viruses [2]. The rice tungro bacilliform virus (RTBV) is a double-stranded deoxyribonucleic acid (DNA) virus from the family Caulimoviridae, of the genus* Tungrovirus* [3], and the rice tungro spherical virus (RTSV), a single-stranded ribonucleic acid (RNA) virus from the family Sequiviridae, of the genus* Waikavirus* [4]. RTSV has a single-strand polyadenylated RNA genome of about 12 kb that encodes a single large open reading frame (ORF). The structure of RTSV particles is spherical or icosahedral with a diameter of 30–33 nm. Its capsid comprises three coat proteins, namely, CP1, CP2, and CP3 [5]. On the other hand, RTBV has a circular double-stranded DNA genome of 8 kb that encodes four ORFs. RTBV has a bacilliform structure with width and length of 38 nm × 200 nm, respectively [6]. The symptoms and severity of this disease depend on these two viral agents. If rice is coinfected by both of the viruses, it will show the typical severe symptoms of yellow-orange leaf discoloration, plant stunting, and reduced yield [7]. On the other hand, if rice is infected only with RTBV, it shows milder symptoms. In contrast, rice plants will show no symptoms if they are infected only with RTSV [8].

Generally, except in advanced laboratories, RTD is commonly identified by visual observation of the symptoms. However, visual identification based on the symptoms alone is not reliable and often confused with other diseases and nonpathogenic disorders that can cause similar symptoms [9]. Conventionally, insect transmission assays had been used to identify tungro-infected rice plants; however, these assays are not necessarily specific for tungro and are laborious and time-consuming [10]. Currently, different molecular techniques such as restriction fragment-length polymorphisms (RFLP) [11], PCR [12], multiplex RT-PCR [13], RT-LAMP [14], and real-time PCR [15] are used in detecting and screening for RTD. Although detection by PCR and the reverse transcriptase PCR are considered the most rapid and sensitive techniques to detect low levels of RTBV and RTSV, respectively [16], the application of molecular techniques in detecting RTD may not be appropriate when screening for a large number of field samples, for it can be costly and labor intensive. Detection by serological assays had also been reported which are shown to be relatively more specific, sensitive, and reliable [17]. In 1985, Bajet and colleagues [18] had developed a double antibody sandwich (DAS) ELISA for detection of RTBV and RTSV separately in infected plants propagated in greenhouse. This technique was used in the Philippines in the 1990s to survey or monitor tungro spread throughout the Philippines [19]. However, the technique was not widely used in rice-growing countries due to limitation on the availability of reliable sera and laboratory facilities. Nath and colleagues [20] had attempted to produce high titre polyclonal antisera against RTBV and RTSV for use in simple rapid diagnostic tests. The study reported that the polyclonal antisera worked well in the DAS-ELISA; however, the multiwell plate based ELISA may not be practical in many situations where the facilities to perform an ELISA may not be available. We report here the development of a simplified ELISA from what was previously described and the modification of the simplified ELISA onto a dot-blot assay platform which is relatively cheaper and does not require much knowledge and skills to perform.

## 2. Materials and Methods

### 2.1. Samples

The source of RTBV and RTSV used in this study was maintained as a combination of both viruses in rice variety, Taichung Native 1 (TN1). Infected and healthy field samples were collected and provided by ARC, Tuaran, Department of Agriculture, Sabah, and by ARC, Semongok, Department of Agriculture, Sarawak. Use of plant materials in this study was regulated and in compliance with guidelines as stated in Plant Quarantine Act, 1976, Section 14(c) (Plant Quarantine Act, 1976) [21].

### 2.2. Virus Purification

The viruses were purified based on a method described elsewhere [2] that gives a rapid preparation of unseparated viruses, with slight modification. For this purpose, 300 grams of infected TN1 leaves was frozen and blended in 4 mg/ml of cold Buffer A (0.1 M sodium citrate, pH 6; 0.01 M EDTA). Next, 50,000 units of Celluclast (Sigma, St. Louis, MO, USA) were added and the mixture was incubated at 40°C in an incubator shaker for 3 hours before being filtered through two layers of muslin cloth. After another 3 hours at 40°C, the filtrate was centrifuged at 15,000 ×g for 15 minutes at 4°C (Beckman, J-Avanti). The supernatant was collected and added with 7% (v/v) polyethylene glycol (PEG), 0.2 M sodium chloride, and 1% (v/v) Triton X-100. The mixture was centrifuged at 16,000 ×g for 35 minutes at 4°C after stirring at room temperature for one hour. The collected pellet was resuspended in 6 ml of Buffer A and incubated overnight at 4°C. The next day, the mixture was centrifuged at 8000 ×g for 15 minutes at 4°C. The supernatant was collected, laid onto a 10% sucrose cushion, and centrifuged in a Beckman SW 41 Ti rotor at 36,000 rpm for 2.5 hours at 4°C (Beckman Optima, XL-100 K). The collected pellet was resuspended in 1 ml of Buffer A and incubated overnight at 4°C. The next day, the mixture was centrifuged at 8000 ×g for 15 minutes at 4°C. The supernatant was collected, laid onto a 10 to 50% sucrose density gradient, and centrifuged in a Beckman SW41 Ti rotor at 40,000 rpm for 3 hours at 10°C. The opalescent bands formed were collected, diluted in Buffer B (0.1 M sodium citrate, pH 7), and centrifuged in a Beckman SW41 Ti rotor at 40,000 rpm for 4 hours at 4°C. The collected pellet was resuspended in 1.2 ml of Buffer C (0.01 M phosphate buffer, pH 7.4), centrifuged at low speed to collect the supernatant, and aliquoted into a few tubes of 1.5 ml microcentrifuge tube.

### 2.3. Preparation of Rabbit Antiserum

Rabbit immunization schedule was based on Nath and colleagues [20], as previously mentioned, with slight modification. The rabbit was prebled prior to immunization. The experimental rabbit was injected with one volume of 0.2 mg/ml of purified tungro viruses in 0.01 M phosphate buffer, pH 7.4, and one volume of Freund's complete adjuvant through the subcutaneous route. Two similar doses were injected at weeks 2 and 4. After the third injection, booster injections with one volume of 0.2 mg/ml of purified tungro viruses added with one volume of Freund's incomplete adjuvant were injected into rabbit at weeks 6 and 8. Ear bleed was carried out weekly after the last booster. The experiment was conducted in compliance with Veterinary Public Health Ordinance, 1999 [22], in the State of Sarawak, Malaysia, under close supervision of State Veterinary.

### 2.4. Lysates and Leaf Sap Preparations

Lysates from healthy and infected TN1 were processed in bulk and were used as negative and positive control lysates in this study. These lysates were prepared as 0.1 mg/ml stocks. Each stock was prepared by homogenizing 1 part of leaf with 10 parts (w/v) of 1x PBS, pH 7.4, incubated at 40°C for 30 mins and clarified by centrifugation. Leaf saps were also prepared from the healthy and infected TN1. Leaf sap was prepared by grinding 3 pieces of 1 cm^2^ of leaf in a 1.5 ml microcentrifuge tube filled with 500 *μ*l of 1x PBS, pH 7.4. The homogenate was then incubated at 40°C for 10 mins and ground again before being subjected to centrifugation at 10,000 ×g for 5 mins.

### 2.5. ELISA Procedure

The indirect ELISA was performed in 96-well maxisorp immunoplates (Nunc). Paired rows of wells were coated with 100 *μ*l/well of 1 *μ*g of positive and negative lysates or 1 : 40 dilution of the test leaf saps in carbonate-bicarbonate, coating buffer (pH 9.6), for an overnight incubation at 4°C. The next day the plates were washed once with 300 *μ*l/well of 1x PBST (1x PBS with 0.05% Tween 20, pH 7.2) wash buffer. Subsequently, the plates were blocked with 200 *μ*l/well of 1% casein/PBS, pH 7.2 (CPBS), blocking buffer for 2 hrs at room temperature. The plates were subjected to 3x washes at 1 min interval. Then 100 *μ*l/well of the tungro antiserum and control serum (1 : 500 in CPBS) was added to the wells. Plates were incubated at 4°C overnight and were subjected to 6x washes the next day. A volume of 100 *μ*l/well of commercial HRP-conjugated swine anti-rabbit immunoglobulins (1 : 2000 dilution in CPBS) was added for one-hour incubation followed by another 6x wash as previously mentioned. Color was developed by adding 100 *μ*l/well of chromogenic substrate 0.05 mg/ml of o-phenylenediamine in citrate buffer, pH 5, with 0.01% H_2_O_2_. After 30 mins, the reaction was stopped with 50 *μ*l/well of 2.5 M sulphuric acid, stopping buffer. Absorbance value was read at 492 nm using a multimode microplate reader (Biotek). Each of the test samples was tested in triplicate and average absorbance values were statistically analyzed.

### 2.6. ELISA Cut-Off Point Determination

To determine the optimal cut-off value for the indirect ELISA, 59 known healthy rice plant samples were tested in the indirect ELISA using the optimized parameters. The mean and SD of the dataset were calculated and the 95% confidence interval (CI) of the mean was chosen as the cut-off value. The upper limit of the 95% CI was calculated based on the t distribution from the 59 samples based on standard formula: [Upper limit = *M* + *Z*.95*σM*, where *M* = samples mean; *Z*.95 = is the number of standard deviations extending from the mean of a normal distribution required to contain 0.95; *σM* = standard error of the mean] or equivalent to mean + 2SD [23]. Test samples were considered positive if the OD ≥ mean + 2SD and considered negative if the OD < mean + 2SD.

### 2.7. Dot-Blot Assay Procedure

The dot-blot assay was done based on an in-house assay described elsewhere [24] with modification. Briefly, 2 *μ*l control lysates and leaf saps of test samples were spotted at 1 : 2 dilution in PBS, pH 7.2. The NCMs were air-dried and blocked in 5% nonfat skimmed milk/PBS, pH 7.2 (NFSM-PBS), for 30 mins. The membranes were then washed 3 times at 10 mins interval with 1x PBS, pH 7.2. The membranes were then incubated with the tungro antiserum at 1 : 100 dilution with an overnight incubation with rocking. The next day, the membranes were subjected to 3 times wash with 1x PBS for 30 minutes each at 10 minutes interval prior to 2-hour incubation with HRP-conjugated swine anti-rabbit immunoglobulins (1 : 1000). Subsequently, the membranes were washed and color was developed with chromogenic substrate 4-chloro-1-naphtol plus H_2_O_2_ for 30 mins in the dark.

### 2.8. PCR

Viral DNA was extracted from healthy and infected plants using High Pure Viral Nucleic Acid Extraction Kit (Roche Diagnostics GmbH, Germany). Prior to the extraction protocol, three pieces of 1 cm^2^ of leaves were ground in liquid nitrogen, added with 1x TE buffer, pH 8.0, and binding buffer, and the homogenate was clarified by centrifugation, 13,000 rpm, 5 mins before transferring clear supernatant to the filter tube and collection tube for further processing according to the manufacturer's protocol. The PCR for both RTBV and RTSV was performed using published primers and protocol based on Dasgupta and colleagues [12] and Periasamy et al. [13], as previously mentioned.

## 3. Results and Discussion 

### 3.1. PCR

In this study, a total of 99 samples that consist of infected and healthy plants were tested. The results of the RTBV and RTSV PCR according to published protocols as previously mentioned were recorded and used as comparison to both the results of the ELISA and dot-blot assay. Although initially all samples were tested for both RTBV and RTSV, the RTSV PCR, however, was less sensitive and inconsistent for the detection of the positive samples compared to the RTBV PCR (see Supplementary Materials 1 and 2 in Supplementary Material available online at https://doi.org/10.1155/2017/3608042). Therefore, finally, for the purpose of this study, the comparison was done with the results of the RTBV PCR only.

### 3.2. Indirect ELISA

**Figure 1:**
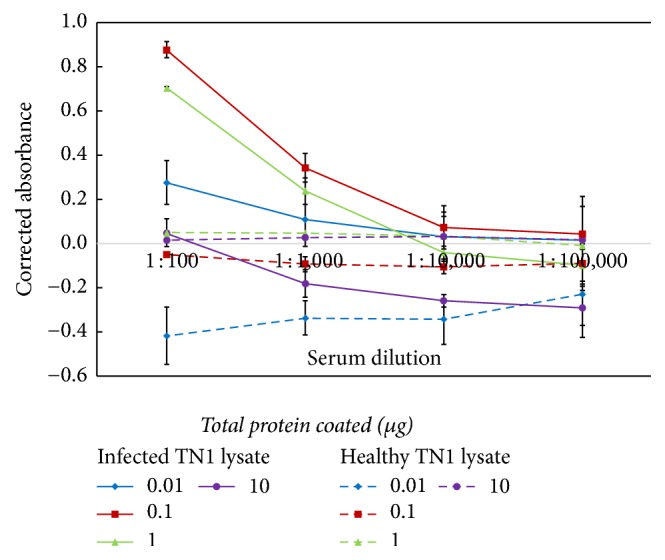
Optimization of the amount of antiserum and lysate to be used in the indirect ELISA. The amount of control lysate coated and antiserum dilution used were tested at 10-fold ranging from 10 to 0.01 *μ*g concentration and from 1 : 100 to 1 : 100,000 dilution, respectively, in a checkerboard titration.

A checkerboard titration to determine the optimal dilution factor for both the antiserum and antigen coating was performed. Several concentrations and dilutions of the positive and negative lysate coated and the tungro antiserum were tested. The amount of lysate coated was tested at different concentrations ranging from 10 to 0.01 *μ*g at a 10-fold serial dilution. The dilution of the tungro antiserum to be used were tested at different dilutions ranging from 10^2^ to 10^5^. The prebleed serum was used as the control serum in the assay. The commercial HRP-conjugated swine anti-rabbit immunoglobulins (Dako) was used as the secondary antibody in the assay. The titration result is as presented in Figure 1. Based on the optimization, lysate coated at 0.1 *μ*g total protein gives the best differences in the OD between the positive and negative lysates. When coated at this concentration, the antiserum showed a distinct OD reading between the negative and positive control lysates with the highest at 1 : 500 dilution (OD > 0.5). For this assay, the optimum dilution of the antiserum was established at 1 : 500 dilution and positive and negative lysates control were determined to be used at 0.1 *μ*g for the indirect ELISA. The positive and negative lysates were prepared in bulk and were used as the standards to develop the assays.

To determine the cut-off point for the assay, 59 known healthy samples were analyzed in the assay and the upper limit of mean + 2SD value; 0.457 was determined as the cut-off point. All test samples values ≥ 0.457 were considered positive for RTD. To validate the assay, 99 field samples collected from various fields from the states of Sabah and Sarawak were tested. The PCR and ELISA results of the 99 samples were compared (Supplementary Material 1) and the analysis is as summarized in Table 1. The indirect ELISA correctly identified 39 of the 40 positive samples with only 1 sample being misclassified (false negative). Out of the 59 negative samples only 2 samples were misclassified (false positive). The performance of the indirect ELISA in comparison to the established PCR was evaluated based on the diagnostic parameters shown in Table 2. The sensitivity [Se = TP/(TP + FN); where TP = true positive, FN = false negative] and specificity [Sp = TN/(FP + TN); where TN = true negative, FP = false positive] of the simplified indirect ELISA was calculated at 95% confidence interval [25]. The data shows a sensitivity and specificity of 0.98 (CI 95%: 0.82 to 0.99) and 0.97 (CI 95%: 0.87 to 0.99), respectively. In addition to the sensitivity and specificity, the likelihood ratios at 95% CI were also determined [26]. The likelihood ratios described the odds favoring the disease given a particular test. The positive and negative likelihood ratios (Table 2) for the test were 28.67 (CI 95%: 7.359–112.414) and 0.026 (CI 95%: 0.004–0.179), respectively.

### 3.3. Dot-Blot Assay

**Figure 2:**
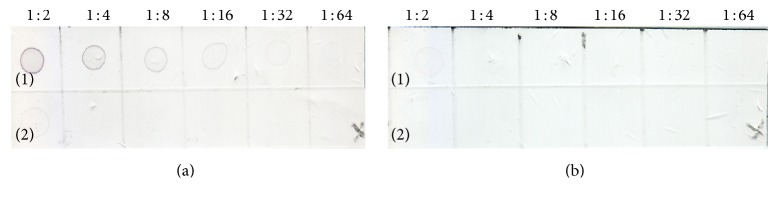
Control lysates titration. Top row spotted with positive control lysate diluted twofold (from 1 : 2 to 1 : 64) with 1x PBS pH 7.4. Bottom row spotted with negative control lysate subjected to the same treatment. (a) NCM probed with positive antiserum while membrane in (b) NCM probed with negative antiserum.

**Figure 3:**
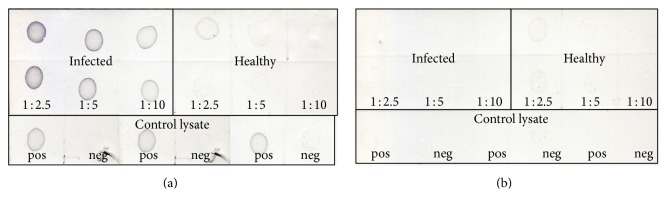
Leaf sap titration. Both membranes were spotted with the same samples and in the same order. The first three samples from the left were leaf sap from infected TN1 diluted twofold (1 : 2.5, 1 : 5, and 1 : 10) with 1x PBS pH 7.4. The last three samples were leaf sap from healthy TN1 subjected to the same treatment. Membrane in (a) was probed with positive antiserum while membrane in (b) was probed with negative antiserum. The last row was spotted with control lysates, positive and negative diluted 1 : 4 with 1x PBS.

**Figure 4:**
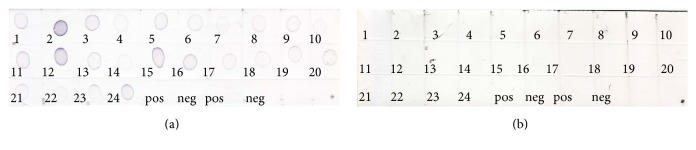
Reactivity of 24 known RTD-infected samples with the generated antisera. Both membranes were spotted with the same samples and in the same order. (a) The membrane was probed with positive antiserum. (b) The membrane was probed with negative antiserum. Positive and negative lysates were dotted in duplicate at the end of each membrane.

In the dot-blot assay, the multiwell plate based indirect ELISA was modified and the antigen was directly dotted onto a nitrocellulose membrane (NCM). The control lysates were tested at dilution ranging from 1 : 2 to 1 : 64 in a 2-fold serial dilution in PBS, pH 7.2 (Figure 2), and samples from the leaf saps preparation were tested at dilution ranging from 1 : 2 to 1 : 10 at a 2-fold serial dilution in PBS, pH 7.2 (Figure 3). The dilution of the antiserum was tested at a range of 1 : 100 to 1 : 400 while the commercial conjugate used was tested at 1 : 1000 and 1 : 2000 for the best dilution with minimal background. The optimization of the antiserum adsorption to healthy plant components was also carried out to reduce the background in the assay. The adsorption was done by incubating one volume of antiserum to two volumes of healthy plant lysate at 37°C for an overnight incubation followed by clarification by centrifugation. The same set of samples tested for in the indirect ELISA were also tested in the dot-blot assay. The reactivity of the first 24 samples is as presented in Figure 4. The dots with intensity similar or greater than the positive control dot were scored as positive otherwise scored as negative

The performance of the dot-blot assay as compared to the results of the established PCR was compared (Supplementary Material 1) and the analysis of the result is as presented in Table 3. The sensitivity and the specificity of the assay were determined as previously shown (Table 2). The data shows a sensitivity and specificity of 0.98 (CI 95%: 0.85 to 0.99) and 0.86 (CI 95%: 0.74 to 0.94), respectively. In addition, the likelihood ratios at 95% CI were also determined. The positive and negative likelihood ratios for the test were 7.19 (CI 95%: 3.77–13.72) and 0.030 (CI 95%: 0.004–0.201), respectively.

## 4. Conclusion

RTD is one of the most damaging diseases of rice in Southeast Asia, including Malaysia. The disease had been detected in Malaysia since 1930s [27]; however, for the two states in East Malaysia (Sabah and Sarawak), which are the focus of this study, RTD have been reported much later. Sabah had reported suspected RTD outbreak as early as 1970s (personal communication) although it was initially declared free of RTD; Sarawak reported her first incidence of RTD in 2012 [28]. Due to its destructiveness, an early detection of the disease is important to prevent reduction in yield of rice production and to offer a better solution to farmers. The current practice of detecting RTD by visual observation had been shown to be unreliable and often mistakes RTD for other diseases or disorders with similar symptoms. Although molecular techniques such as PCR have been widely used to detect RTD, the method is not cost-effective, especially, for screening large number of samples collected from fields for survey and monitoring of RTD. Therefore, in this study, we had developed two simple serological assays, a plate based indirect ELISA and a membrane based dot-blot assay. The results show that both assays have high sensitivity and specificity for RTD when compared to the established PCR method. Both assays showed a sensitivity of about 98%. While the specificity for the ELISA was also determined at 98%, the dot-blot assays have slightly lower estimation at around 86%. Both assays also showed a very good agreement compared with the established PCR method with a Kappa index of 0.92 and 0.80 for the ELISA and the dot-blot, respectively. These values fall under the Kappa interpretation of almost perfect agreement (0.81–0.99) and substantial agreement (0.61–0.80) for the ELISA and the dot blot, respectively, where Kappa index of 1 is perfect agreement [29]. Although the indirect ELISA performed much better than the dot-blot assay, the latter has advantages to the former where no specific equipment is required and it is easy and much simpler to perform. The ELISA on the other hand has an advantage of being more specific than the dot-blot assay. Therefore each assay can complement the other, where the dot-blot assay can be performed as the preliminary screening method that can be done at a closer vicinity of the paddy fields and the ELISA can be used where laboratory facility is available. However both assays have a great advantage to PCR, where they are more cost-effective for screening larger sample numbers. In conclusion, the high sensitivity and specificity of the two assays reported in this study show great potential to be used as assays for monitoring and to survey the spread of RTD in paddy fields in remote areas, such as in Sabah and Sarawak, the two states in East Malaysia.

## Supplementary Material

Supplementary Material 1: The PCR, dot-blot and ELISA results of all the 99 samples tested in this study.
Supplementary Material 2: The RTBV and RTSV PCR results using published and in-house designed primer sets.

## Figures and Tables

**Table 1:** Comparison of RTD results by simple indirect ELISA and the reference PCR assay.

Indirect-ELISA results	PCR results (number of samples)		
	Positive	Negative	Total number of samples
Positive	39^a^	2^b^	41
Negative	1^c^	57^d^	58

Total	40	59	99

^a^True positive (TP); ^b^false positive (FP); ^c^false negatives (FN); ^d^true negative (TN).

**Table 2:** Calculation of diagnostic parameters of the indirect ELISA and the dot-blot assay.

Diagnostic parameters	Estimated value	95% confidence interval	
		Lower limit	Upper limit
*Indirect ELISA*			
Sensitivity (Se)	0.975	0.853	0.998
Specificity (Sp)	0.966	0.872	0.994
Likelihood ratio +ve (LR+)	28.67	7.359	112.414
Likelihood ratio −ve (LR−)	0.0258	0.004	0.179
*Dot-blot*			
Sensitivity (Se)	0.975	0.852	0.998
Specificity (Sp)	0.864	0.745	0.935
Likelihood ratio +ve (LR+)	7.190	3.768	13.721
Likelihood ratio −ve (LR−)	0.030	0.004	0.201

Se = [TP/(TP + FN)], Sp = [TN/(FP + TN)], LR (+) = [Se/(1 − Sp)], LR (−) = [(1 − Se)/Sp].

**Table 3:** Comparison of RTD results by dot-ELISA and the reference PCR assay.

Dot-blot results	PCR results (number of samples)		
	Positive	Negative	Total
Positive	39^a^	8^b^	47
Negative	1^c^	51^d^	52

Total	40	59	99

^a^True positive (TP); ^b^false positive (FP); ^c^false negatives (FN); ^d^true negatives (TN).
