# Supplementary material for: Development of an Indirect ELISA and Dot-Blot Assay for Serological Detection of Rice Tungro Disease
Source: Biomed Res Int. 2017 Oct 22;2017:3608042. doi: 10.1155/2017/3608042 (PMC5671674; doi:10.1155/2017/3608042)
Supplement: Supplementary file 1 — Supplementary Material 1: The PCR, dot-blot and ELISA results of all the 99 samples tested in this study. Supplementary Material 2: The RTBV and RTSV PCR results using published and in-house designed primer sets. [file 3608042.f1.pdf]

Supplementary Material 1: The PCR, dot-blot and ELISA results of all the 99 samples tested in this study

No	Sample ID	RTBV PCR result	RTSV PCR result	Dot blot result	Indirect ELISA result	
					Corrected OD value	
					Average*	SD
1	IP1	+	-	+	0.5015	0.0276
2	IP2	+	-	+	0.5070	0.0283
3	IP3	+	+ (weak)	+	1.4660	0.0382
4	IP4	+	+	+	0.6295	0.0262
5	IP5	+	+	+	0.7960	0.0820
6	IP6	+	-	+	1.0795	0.0516
7	IP7	+	+	+	1.1035	0.1831
8	IP8	+	-	-	1.1740	0.0085
9	IP9	+	-	+	1.5855	0.1221
10	IP10	+	-	+	1.8130	0.1047
11	IP11	+	+ (weak)	+	2.3855	0.0969
12	IP12	+	+ (weak)	+	2.4920	0.1047
13	IP13	+	-	+	2.8285	0.3981
14	IP14	+	+ (weak)	+	0.5215	0.0134
15	IP15	+	+	+	0.6935	0.0955
16	IP16	+	+	+	0.7695	0.0813
17	IP17	+	-	+	0.8470	0.1796
18	IP18	+	-	+	0.9125	0.2821
19	IP19	+	-	+	1.0525	0.1025
20	IP20	+	-	+	1.1965	0.0177
21	IP21	+	-	+	1.2130	0.0806
22	IP22	+	-	+	1.3445	0.0813

23	IP23	+	-	+	1.5555	0.1185
24	IP24	+	-	+	2.0150	0.1031
25	IP25	+	+	+	0.4690	0.0707
26	IP26	+	-	+	0.6160	0.0311
27	IP27	+	+	+	0.6940	0.0071
28	IP28	+	-	+	0.7035	0.0007
29	IP29	+	-	+	0.7065	0.1336
30	IP30	+	-	+	0.7510	0.0184
31	IP31	+	+	+	0.9010	0.0057
32	IP32	+	+ (weak)	+	1.2755	0.0615
33	IP33	+	+	+	1.3490	0.0057
34	IP34	+	-	+	1.4665	0.0304
35	IP35	+	-	+	1.4750	0.0665
36	IP36	+	-	+	1.7465	0.2821
37	IP37	+	+ (weak)	+	1.7715	0.2623
38	IP38	+	-	+	2.7440	0.0594
39	IP39	+	-	+	2.9545	0.0742
40	IP40	+	+	+	0.3410	0.0269
41	HP1	-	-	-	0.2765	0.0488
42	HP2	-	-	-	0.2965	0.0318
43	HP3	-	+ (weak)	-	0.2750	0.0750
44	HP4	-	-	-	0.2385	0.0742
45	HP5	-	-	-	0.3085	0.1266
46	HP6	-	-	-	0.2395	0.1096
47	HP7	-	-	-	0.3960	0.0014
48	HP8	-	-	-	0.2890	0.0552
49	HP9	-	-	-	0.3410	0.0594

50	HP10	-	-	+	0.3665	0.0403
51	HP11	-	-	-	0.3190	0.0891
52	HP12	-	-	-	0.3560	0.0594
53	HP13	-	-	+	0.2695	0.1831
54	HP14	-	-	-	0.3550	0.0113
55	HP15	-	-	-	0.2360	0.0552
56	HP16	-	-	-	0.3010	0.0014
57	HP17	-	-	-	0.1935	0.0078
58	HP18	-	-	+	0.2285	0.0544
59	HP19	-	-	-	0.2620	0.0184
60	HP20	-	-	-	0.1595	0.0304
61	HP21	-	-	-	0.3590	0.0537
62	HP22	-	-	-	0.3640	0.0325
63	HP23	-	-	-	0.2000	0.0622
64	HP24	-	-	-	0.1925	0.0106
65	HP25	-	-	+	0.2975	0.0262
66	HP26	-	-	-	0.3205	0.0262
67	HP27	-	-	-	0.2590	0.0028
68	HP28	-	-	-	0.2505	0.0035
69	HP29	-	-	-	0.2410	0.0396
70	HP30	-	-	-	0.4525	0.1435
71	HP31	-	-	-	0.4165	0.0191
72	HP32	-	-	-	0.3455	0.0771
73	HP33	-	-	-	0.2785	0.0940
74	HP34	-	-	-	0.3405	0.0163
75	HP35	-	-	-	0.3705	0.0262

76	HP36	-	-	-	0.3475	0.0460
77	HP37	-	-	-	0.3890	0.0283
78	HP38	-	-	-	0.3000	0.0057
79	HP39	-	-	-	0.1510	0.0071
80	HP40	-	-	-	0.3320	0.0354
81	HP41	-	-	+	0.3915	0.0276
82	HP42	-	-	-	0.2720	0.0141
83	HP43	-	-	-	0.2235	0.0148
84	HP44	-	-	-	0.2665	0.0205
85	HP45	-	-	-	0.2885	0.0346
86	HP46	-	-	-	0.3350	0.0325
87	HP47	-	-	-	0.2215	0.0078
88	HP48	-	-	-	0.2550	0.0184
89	HP49	-	-	-	0.2810	0.0750
90	HP50	-	-	-	0.4695	0.0346
91	HP51	-	-	-	0.5275	0.0219
92	HP52	-	-	+	0.3015	0.1379
93	HP53	-	-	-	0.2850	0.0042
94	HP54	-	-	-	0.3310	0.0042
95	HP55	-	-	-	0.2770	0.0283
96	HP56	-	-	-	0.3105	0.0573
97	HP57	-	-	-	0.2445	0.0460
98	HP58	-	-	+	0.3920	0.0509
99	HP59	-	-	+	0.4525	0.0361

IP = infected plant sample; HP = healthy plant sample; + = positive result; - = negative result; * = Average of two corrected OD values

Note:

1. Based on RTBV PCR as described by Dasgupta et al, 1996.

2. Combination of RTSV PCR done based on Perisamy et al, 2006 and in-house primer set (unpublished)

All the samples were tested by the three different assays. The results from the dot-blot and ELISA were compared to the result from the established PCR assay. The data generated from this table were used to tabulate the 2x2 table (Table 1 and Table 3) shown in the main manuscript. The OD from the ELISA was expressed as an average from a duplicate test.

Supplementary Material 2: The RTBV and RTSV PCR results using published and in-house designed primer sets.

RTBV PCR

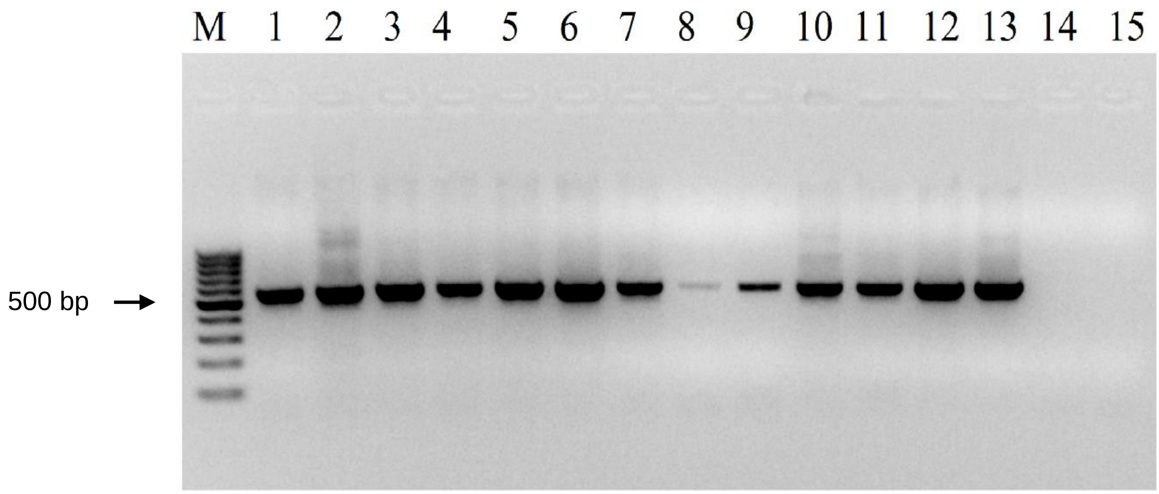

(a)

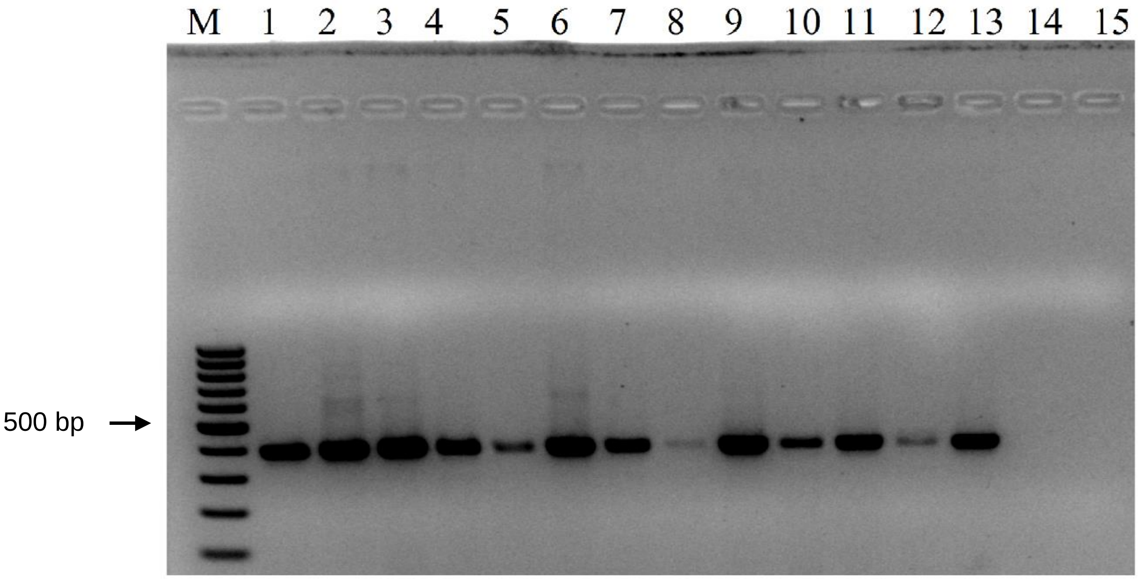

(b)

Figure 1: Agarose gel electrophoresis analysis of PCR products to detect RTBV amplified using (a) modified method from Dasgupta *et al.* (1996) (b) in-house designed primers to ORF1 region; ~ 400 bp (unpublished primers). Lane M: 100 bp DNA marker (Thermo Scientific); Lane 1 to 12: samples (representative of the twelve first samples as listed in Supplementary Material 1); Lane 13: Positive control (infected TN1 lysate); Lane 14: Negative control (Healthy TN1); Lane 15: water control.

RTSV PCR

We tested the multiplex PCR from Periasamy *et al*. (2006) and only RTBV was amplified. We also tested the RTSV primers from Periasamy *et al*. (2006) in a single RTSV amplification and obtained inconsistent results as shown in the Figure 2 below where RTSV PCR was conducted on infected samples. In addition, we obtained multiple bands besides the targeted band at approximately 1.1 kbp as mentioned by Periasamy *et al.* (2006). Thus, we design our own in-house primer set (unpublished primer) to re-attempt the RTSV PCR and although with this set of primer a single band was observed at the expected size (700 bp) the sensitivity is still lower than the RTBV PCR.

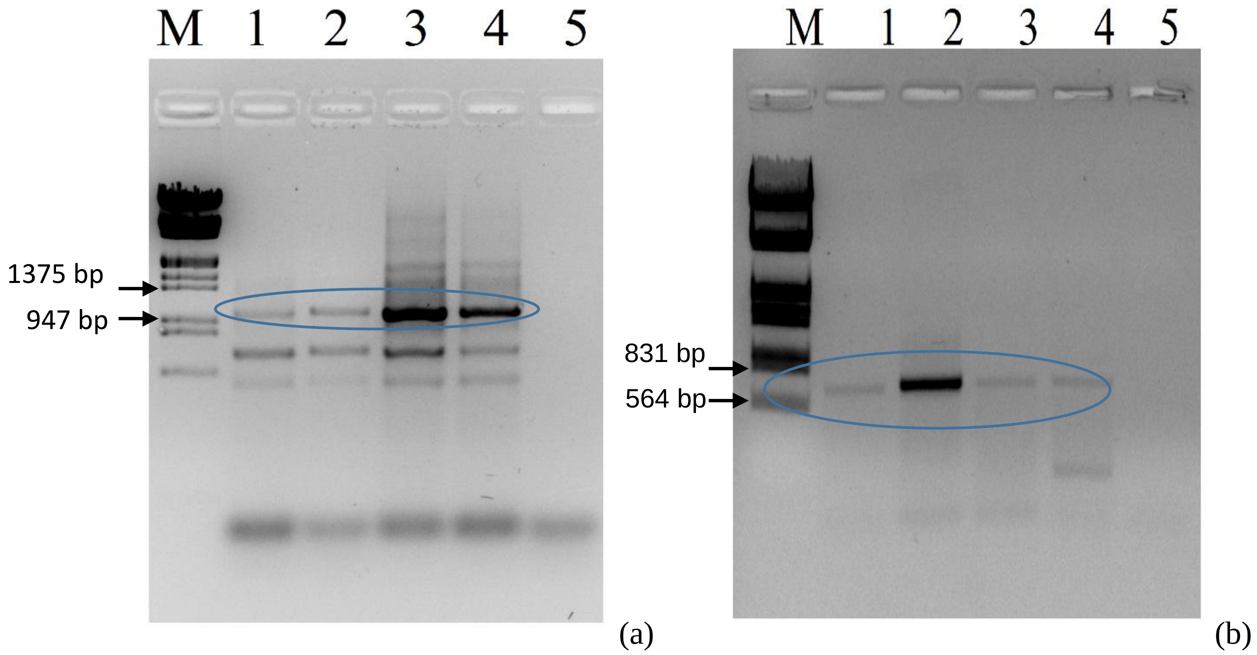

Figure 2: Agarose gel electrophoresis analysis of PCR products to detect RTSV amplified using (a) modified method from Perisamy *et al.* (2006) (b) in-house designed primers (to CP1 and CP2 regions of RTSV; ~700 bp). Lane M: Lambda DNA/ Eco R1/ Hind III marker (Thermo Scientific); Lane 1 to 4: Infected samples (representative of the first 4 samples as listed in Additional File 1); Lane 5: water control

References:

1. Dasgupta I, Das BK, Nath PS, Mukhopadhyay S, Niazi FR, Varmac A. Detection of rice tungro bacilliform virus in field and glasshouse samples from India using the polymerase chain reaction. J Virol Methods. 1996; 58: 53-58.
2. Periasamy M, Niazi FR, Malathi VG. Multiplex RT-PCR, a novel technique for the simultaneous detection of the DNA and RNA viruses causing rice tungro disease. J Virol Methods. 2006; 134: 230-236.
